# Apicomplexan parasites are attenuated by low-energy electron irradiation in an automated microfluidic system and protect against infection with *Toxoplasma gondii*

**DOI:** 10.1007/s00436-023-07880-w

**Published:** 2023-05-26

**Authors:** Julia Finkensieper, Florian Mayerle, Zaida Rentería-Solís, Jasmin Fertey, Gustavo R. Makert, Franziska Lange, Joana Besecke, Simone Schopf, Andre Poremba, Ulla König, Bastian Standfest, Martin Thoma, Arwid Daugschies, Sebastian Ulbert

**Affiliations:** 1 Fraunhofer Institute for Cell Therapy and Immunology IZI, Perlickstrasse 1, 04103 Leipzig, Germany; 2 Fraunhofer Institute for Manufacturing Engineering and Automation IPA, Nobelstrasse 12, 70569 Stuttgart, Germany; 3 Institute of Parasitology, Centre for Infection Medicine, Faculty of Veterinary Medicine, Leipzig University, An den Tierkliniken 35, 04103 Leipzig, Germany; 4 Albrecht-Daniel-Thaer Institute, Rudolf-Breitscheid-Str. 35, 04463 Großpösna, Germany; 5 Fraunhofer Institute for Organic Electronics, Electron Beam and Plasma Technology FEP, Winterbergstrasse 28, 01277 Dresden, Germany

**Keywords:** Toxoplasma, Cryptosporidium, Irradiation, Attenuation, Vaccine

## Abstract

Radiation-attenuated intracellular parasites are promising immunization strategies. The irradiated parasites are able to invade host cells but fail to fully replicate, which allows for the generation of an efficient immune response. Available radiation technologies such as gamma rays require complex shielding constructions and are difficult to be integrated into pharmaceutical production processes. In this study, we evaluated for the first time low-energy electron irradiation (LEEI) as a method to generate replication-deficient *Toxoplasma gondii* and *Cryptosporidium parvum*. Similar to other radiation technologies, LEEI mainly damages nucleic acids; however, it is applicable in standard laboratories. By using a novel, continuous, and microfluidic-based LEEI process, tachyzoites of *T. gondii* and oocysts of *C. parvum* were irradiated and subsequently analyzed in vitro. The LEEI-treated parasites invaded host cells but were arrested in intracellular replication. Antibody-based analysis of surface proteins revealed no significant structural damage due to LEEI. Similarly, excystation rates of sporozoites from irradiated *C. parvum* oocysts were similar to those from untreated controls. Upon immunization of mice, LEEI-attenuated *T. gondii* tachyzoites induced high levels of antibodies and protected the animals from acute infection. These results suggest that LEEI is a useful technology for the generation of attenuated Apicomplexan parasites and has potential for the development of anti-parasitic vaccines.

## Introduction

The unicellular parasites *Toxoplasma gondii* and *Cryptosporidium parvum* belong to the Apicomplexa, a phylum of mostly intracellular protozoa which contains several zoonotic pathogens. *Toxoplasma gondii* is one of the most prevalent parasites worldwide and can infect virtually all warm-blooded animals (McAuley, 2014; Opsteegh et al. 2015; Tenter et al. 2000). It is estimated that one-third of the world’s population is chronically infected (Hill and Dubey 2002; Weiss and Dubey 2009). The common transmission routes from animals to humans are ingestion of tissue cysts in undercooked meat, or oocysts in cat feces (Opsteegh et al. 2015; Tenter et al. 2000). *Toxoplasma gondii* is particularly dangerous for immunocompromised people (Dubey et al. 1998). In addition, primary infection during pregnancy can cause fetal abnormalities such as hydrocephalus, microphthalmia, or even stillbirth (Hoffmann et al. 2012; Lopez et al. 2000; Remington 2011; Robert-Gangneux and Dardé 2012). *Cryptosporidium parvum* causes cryptosporidiosis and is a major cause of diarrhea-associated deaths in humans (Kotloff et al. 2013; Lendner et al. 2011; Nguyen et al. 2004). Life-threatening infections occur particularly in immunosuppressed patients and malnourished children (Centers for Disease Control and Prevention 2019). In veterinary medicine, it is a main cause for neonatal diarrhea in calves (Göhring et al. 2014; Lendner et al. 2011) resulting in considerable economic loss (Klein et al. 2008; Thompson et al. 2007).

Available drugs against both parasites often show limited effectiveness or considerable side effects (Blanco-García et al. 2016; Holland and Doggett 2017; Stebbins et al. 2018). Currently there is no vaccine against *C. parvum* available, and the only one against *T. gondii*, Toxovax® for the use in sheep, is live-attenuated by serial in vivo passages (Buxton 1993; Buxton and Innes 1995) and licensed only in a few countries. Attenuation is considered a promising approach for vaccine development against parasites (Pérez Brandan and Basombrío 2012). Attenuated parasites maintain their ability to invade host cells and some of their metabolic activities; however, they are deficient or extremely delayed in replication. This allows for the induction of efficient cellular and humoral immune responses against a broad spectrum of antigens (Minor 2015; Verma and Khanna 2013).

Attenuating a parasite via serial passages in vitro or in vivo is laborious and not always successful. A well-known alternative method is ionizing radiation, so far mostly gamma rays or X-rays. The major effect of ionizing radiation on organisms is the damage of nucleic acids while protein structures remain largely intact (Alsharifi and Müllbacher 2010; Hutchinson 1985). There are several reports on experimental parasitic vaccines generated by ionizing irradiation, including *T. gondii* (Hafez et al. 2020; Hiramoto et al. 2002; Zorgi et al. 2011), *C. parvum* (Jenkins et al. 2004), *Plasmodium falciparum* (Hoffman et al. 2002; James et al. 2022; Oneko et al. 2021), or hookworm larvae (Roh 1968; Xiao et al. 1998).

Despite promising results, most approaches of such radiation-induced anti-parasitic vaccines have so far remained experimental. This is mainly due to the irradiation technologies used. To deliver effective radiation doses, current methods require complex shielding constructions to protect operators and environment from radioactivity. These processes are therefore difficult to incorporate into pharmaceutical production lines (Fertey et al. 2016).

A novel technology to irradiate pathogens in liquid solution is low-energy electron irradiation (LEEI). LEEI consists of electrons with an acceleration voltage up to 300 kilo Volts (kV). LEEI generates low amounts of secondary photon radiation compared to other radiation types which makes it usable in normal laboratories (Fertey et al. 2020). We have previously shown that LEEI efficiently inactivates different viruses and bacteria and provided evidence for the attenuation of the parasite *Eimeria tenella* (Fertey et al. 2016; Thabet et al. 2019). Due to the low penetration depth of the low-energy electrons (Gotzmann et al. 2018; Wetzel et al. 2010), the liquids need to be processed as thin films. Whereas for the treatment of viruses and bacteria processes for automated continuous application of LEEI have been developed, such automated processes do not yet exist for eukaryotic cells, which are more vulnerable to ionizing irradiation and hence tolerate only lower doses (Alsharifi and Müllbacher 2010; Fertey et al. 2020; Finkensieper et al. 2022; Thabet et al. 2019; Walcher et al. 2021). We present here the development of an LEEI process for the treatment of eukaryotic cells and irradiated tachyzoites of *T. gondii* and oocysts of *C. parvum* for the first time with different doses of low-energy electrons. Evidence for reproducible attenuation and the induction of protective immune responses in a mouse model for toxoplasmosis is presented, and discussed in the prospect of developing vaccines.

## Methods

### Cell culture

Vero E6 cells were obtained from DSMZ, HCT-8 cells from CLS (Eppelheim, Germany), and HFF-1 cells from ATCC. Vero E6 and HFF cells were cultured in DMEM (Thermo Fisher Scientific, Waltham, USA) and HCT-8 cells in RPMI1640 (Thermo Fisher Scientific). All media were supplemented with 10 % heat-inactivated FBS (Gibco by Thermo Fisher Scientific) and penicillin (100 U/ml) and streptomycin (100 μg/ml) (Gibco). All cell lines were maintained at 37° C and 5 % CO_2_.

### Parasite cultivation

Tachyzoites of *T. gondii* strains ME49 (type II) and RH-GFP (type I, GFP-expressing strain) (Donald et al. 1996; Sabin 1941) were cultured in Vero E6 cells. For infection, Vero E6 cells were grown until confluency, and washed once with 1x phosphate buffered saline (PBS). The medium was replaced with DMEM with 2 % FCS before tachyzoites were added and the culture was maintained at 37° C and 5 % CO_2_. The medium was exchanged every 2-3 days. Once free tachyzoites were visible in the cell culture supernatant, they were harvested by centrifugation and used for subsequent experiments.

Oocysts of an in-house strain of *C. parvum* were passaged in vivo, isolated, and stored as previously described (Dresely et al. 2015; Najdrowski et al. 2007). Briefly, 4-day-old Holstein–Friesian calves were infected by oral inoculation of 1 × 10^7^ oocysts and their feces was monitored until a sufficient quantity was reached for purification of freshly egressed oocysts. The passages were carried out in accordance with the EU Directive 2010/63/EU for animal experiments and were approved by local authorities (No.: TVV A06/19). Oocysts were suspended in PBS containing penicillin (100 U/ml) and streptomycin (100 μg/ml) and stored at 4° C. They were used for experiments up to 6 months after purification.

### Automated LEEI in a microfluidic system

**Figure 1:**
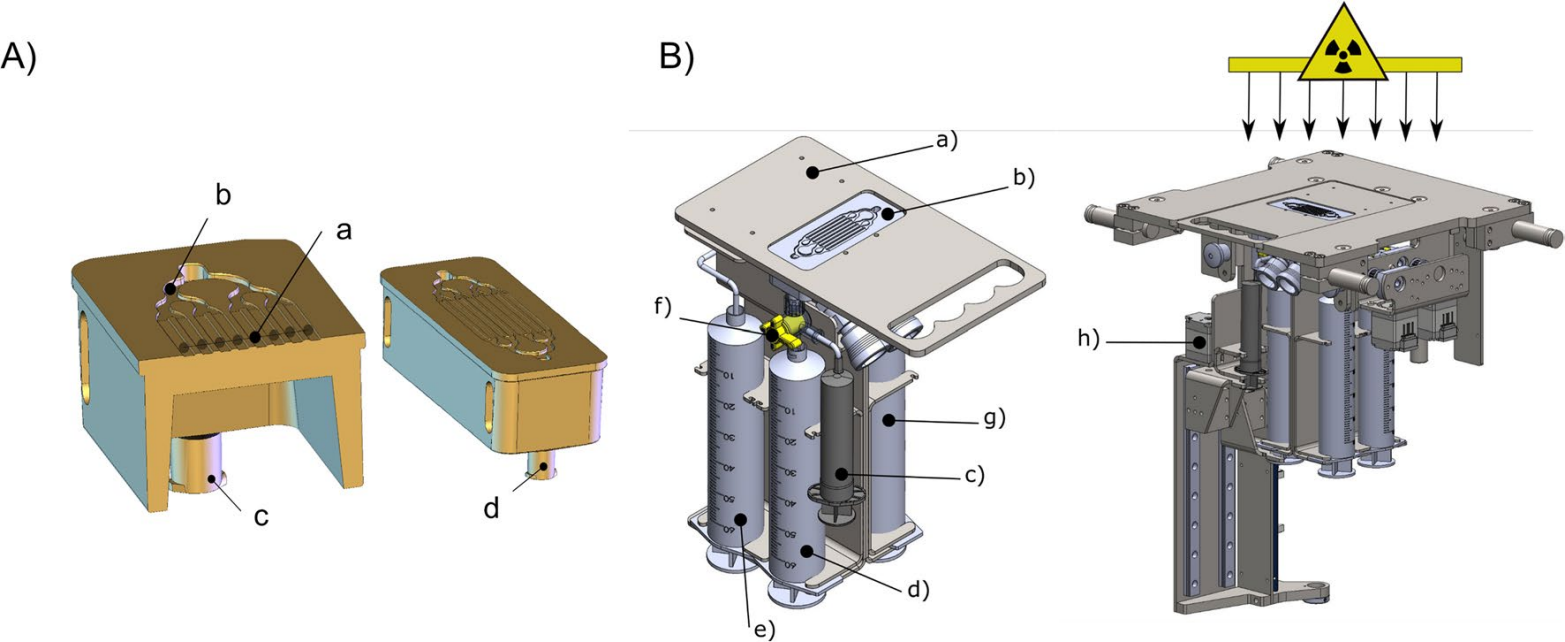
**A** Stainless steel microfluidic chip. Left: (a) Section view on the 8 parallel microfluidic channels with detailed view on (b) deeper flow channels for fluid distribution and (c) the inlet channel with Luer-Lock adapter for connecting tubes. Right: complete view of the microfluidic chip and (d) the outlet channel with Luer-Lock adapter. **B** Left: syringes and chip holder. (a) Main plate with chip recess, (b) stainless steel microfluidic chip, (c) syringe with irradiated sample, (d) waste reservoir, (e) cleaning fluid, (f) valves for control of liquid pathway, and (g) sample reservoir. Right: syringe module with plunger drive and valve control units, and (h) plunger drive for pulling sample from sample reservoir through the chip into the syringe with irradiated sample

Samples were irradiated in a custom-built irradiation device situated at the Fraunhofer Institute for Cell Therapy and Immunology (Fertey et al. 2020). This electron beam device can be equipped with different modules build as research prototypes that enable automated LEEI of liquid solutions. The prototype used in this study centers around a microfluidic chip (MFC) made of stainless steel with 8 parallel channels milled into it (Fig. 1A). Due to the low penetration depth of LEEI of approx. 200 μm, the channel depth was set to 180 μm for the MFC in the irradiation area. To close the system during LEEI, the MFC was sealed with a self-adhesive, thermo-resistant polyester film (Brand, Wertheim, Germany). The liquid reservoirs in this setup were four disposable 60-ml syringes filled with different solutions relevant for the process and inserted into a metal carrier (Fig. 1B). Two were filled with either a cleaning fluid (70 % (v/v) ethanol) (Fig. 1B-e) or sterile buffer. The third syringe served as a waste reservoir (Fig. 1B-d) and the fourth contained the sample to be irradiated (Fig. 1B-g). The reservoirs were connected with disposable plastic parts. The path of the fluids was controlled by three-way valves (Fig. 1B-f), whose positions were automatically adjusted by the control software. During the process, a 10-ml syringe (Fig. 1B-c) was drawn up with a spindle drive (Fig. 1B-h) resulting in a negative pressure to draw the liquid from the sample reservoir through the MFC. It first flowed through the inlet channels into a deeper area and was then distributed to the eight parallel microfluidic channels on which the electron beam was focused on. The irradiated liquid was collected in the 10-ml syringe.

To investigate the flow velocities in the channels of the MFC, a particle image velocimetry method was used. Fluorescent particles (20 μm Fluoresbrite® YG Microspheres) were used as tracer particles, which were sucked through the channels. Their movement through the MFC was recorded simultaneously in all eight parallel channels using a camera (Basler acA2040-90uc) and an optical filter system. Subsequently, the individual particles were extracted from the captured images, the trajectories were linked, and their maximum velocity was calculated.

### Low-energy electron irradiation

All irradiation experiments were performed at room temperature with acceleration voltage set to 200 kV and a transportation velocity of 40 mm/s. Irradiation doses were adjusted by regulating the beam current. Based on previous experiments with eukaryotic cells (Fertey et al. 2020; Walcher et al. 2021), a molybdenum slit diaphragm with a gap of 2 mm was used on the electron exit window to reduce the electron flux for irradiation of *T. gondii*. Irradiation of *C. parvum* oocysts, known to be extremely radiation-resistant (Yoon and Yu 2011), was performed without this slit diaphragm. For LEEI treatment, the titers were adjusted to 3 × 10^6^ tachyzoites/ml and 2 × 10^6^ oocysts/ml for *T. gondii* and *C. parvum*, respectively, each in 30 ml PBS/12% trehalose. Controls underwent the same handling procedures without applying LEEI. For both parasites, a variety of amperages were applied to identify a dose range where attenuation could be observed (data not shown), and the most suitable ones were selected for the experiments presented below.

Radiation dosimetry in Gray (Gy) was performed using 2,3,5-tri-phenyltetrazolium chloride (TTC) 0.2% (w/v) in water, as previously described (Fertey et al. 2020; Walcher et al. 2021). Measurements were performed in the kGy range for *C. parvum* and values were adjusted for the irradiation of *T. gondii* by using a reduction factor of 0.05 resulting from the electron flux reduction by the slit diaphragm. The final adaptation of this dosimetry system to the lower dose range (10-500 Gy) is currently in progress.

### Infection assay and quantitative real-time PCR

#### *T. gondii*

Vero E6 cells were seeded in 24-well plates and grown until confluent as described above. Eight wells per sample were infected with 10^4^ tachyzoites each and incubated at 37° C, 5 % CO_2_. As a positive control, cells were infected with non-irradiated parasites (untreated or process control). The negative controls were heat-inactivated samples (70 °C for 10 min) and non-infected cells. The medium was changed every 3 days. At 1, 3, 6, and 10 days post infection (dpi), cells of 2 wells were washed, collected by trypsinization, and stored at −20° C until DNA isolation using the E.Z.N.A.® Tissue DNA Kit (Omega Bio-tek, Norcross, USA) according to the manufacturer’s instructions. Quantitative real-time PCR was performed as previously described (Edvinsson et al. 2006) with the Luminaris Color HiGreen qPCR Master Mix (Thermo Fisher Scientific). For formaldehyde inactivation, tachyzoites of the ME49 strain were harvested from cell culture and centrifuged at 2000 × *g* for 10 min. The pellet was resuspended in 0.5 % (v/v) formalin in PBS and incubated at 4° C for 48 h (Krahenbuhl et al. 1972). To remove the formaldehyde, tachyzoites were centrifuged at 2000 × *g* for 10 min and washed three times with PBS. Inactivation of the sample was analyzed in a cell culture assay as described.

#### *C. parvum*

HCT-8 cells were seeded in 24-well plates and grown until confluent. Infection of the cells was preceded by excystation of sporozoites from the oocysts. A total of 3 × 10^6^ oocysts of each irradiated sample, non-irradiated controls (untreated or process control), and heat-inactivated oocysts (70 °C for 30 min) were centrifuged at 9500 g for 10 min. The pellets were resuspended in 0.8 % (w/v) sodium taurocholate (Sigma-Aldrich, St. Louis, USA) in RPMI 1640 supplemented with 10 % FCS, penicillin (100 U/ml), streptomycin (100 μg/ml), and 2.5 μg/ml Amphotericin B and were incubated for 1 h at 15° C, followed by 1.5 h at 37° C. At this point, full and empty oocysts in a sample were counted in a Neubauer hemocytometer to calculate the excystation rate (percentual number of empty oocysts from total number). The samples were passed through membrane filters with a 5-μm pore size to separate the released sporozoites from the oocysts. After centrifugation (3400 g for 10 min), the sporozoites were suspended in 700 μl RPMI 1640 containing penicillin (100 U/ml) and streptomycin (100 μg/ml) and 2,5 μg/ml Amphotericin B. To 6 wells per sample, 100 μl of the sporozoite suspension was added to HCT-8 cells in a 24-well plate and incubated at 37° C, 5 % CO_2_. At 2 h post infection (hpi), 1 and 2 dpi, cells of 2 wells were washed, collected by trypsinization, and stored at −20° C until DNA isolation as described above. Quantitative real-time PCR was performed as previously described (Dresely et al. 2015; Shahiduzzaman et al. 2009) with the Luminaris Color Probe qPCR Master Mix (Thermo Fisher Scientific).

### ELISA

The effect of LEEI on the antigenicity of the parasites compared to the non-treated controls was tested by an indirect enzyme-linked immunosorbent assay (ELISA).

In short, samples of *T. gondii* tachyzoites were coated on clear NUNC 96-well MicroWell™ PolySorp® plates (Thermo Fisher Scientific) in carbonate coating buffer (35 mM NaHCO_3_, 15 mM Na_2_CO_3_, pH 9.6) in a total volume of 100 μl/well over night at 4° C. After washing three times with PBS containing 0.05 % Tween 20 (PBS-T), the plates were blocked with 5 % (w/v) skimmed milk powder in PBS for 2 h at room temperature. The plates were again washed with PBS-T and the primary antibody (*T. gondii* tachyzoites polyclonal from rabbit, antibodies-online, Germany) was added in 100 μl/well, diluted 1:1000 in 5 % skimmed milk in PBS. The plate was incubated for 1 h at room temperature. After another wash step, the secondary antibody (polyclonal goat anti-rabbit IgG-HRP, Dako, Glostrup, Denmark) was added, diluted 1:1000 in 100 μl 5 % skimmed milk in PBS and incubated for 1 h. Following a final wash step, TMB-ELISA substrate (Biozol, Eching, Germany) was added for 30 min and the reaction stopped with H_2_SO_4_ (1 M). Absorbance was measured at 450 nm and 520 nm reference wave length in a standard ELISA reader (Infinite M200, Tecan, Männedorf, Switzerland).

To analyze *T. gondii*-binding antibodies in sera, 1.5 × 10^5^ tachyzoites per well were coated on clear NUNC 96 MicroWell™ PolySorp® plates over night at 4° C. Sera from immunized mice were diluted 1:100 in 5 % (w/v) skim milk in PBS and 50 μl per well was added in duplicates for 2 h at room temperature. *Toxoplasma gondii*-binding IgG were detected with an HRP-conjugated rabbit anti mouse IgG antibody (1:1500, Dako).

Oocysts of *C. parvum* were coated on black NUNC 96-well MicroWell™ MaxiSorp® plates (Thermo Fisher Scientific) with carbonate coating buffer in a total volume of 50 μl/well and dried over night at 37° C. The plates were washed with PBS-T and blocked with 5 % (w/v) skimmed milk powder in PBS for 2 h at room temperature. The primary antibody (*C. parvum* oocyst, polyclonal from goat, Thermo Fisher Scientific) was added in 100 μl/well, diluted 1:500 in 5 % skimmed milk in PBS, and incubated for 1.5 h at room temperature. The plate was washed again and the secondary antibody (polyclonal rabbit anti-goat IgG-HRP, Dako) was diluted 1:10,000, added in 100 μl/well 5 % skimmed milk in PBS, and incubated for 1 h. After washing a last time, the readout was performed using a Berthold Centro XS3 luminometer. Enhanced chemiluminescent substrate (ECL, Thermo Fisher Scientific) was used as the substrate diluted 1:10 in PBS and 100 μl injected into each well. With a delay of 1.5 s, relative light units (RLU) were counted for 1 s.

### Bradyzoite differentiation

The in vitro differentiation of *T. gondii* tachyzoites to bradyzoite cysts was performed as previously described (Mayoral et al. 2020). In short, HFF cells were plated in 24-well cell culture plates until confluency. For infection, the medium was removed and cells were washed once in PBS. 10^5^ tachyzoites of the ME49 strain were added per well in DMEM with 2 % FCS and incubated for 2 h at 37° C and 5 % CO_2_. The supernatant was removed and remaining tachyzoites were washed off twice with PBS. To induce differentiation into bradyzoites, the medium was changed to a differentiation medium (DMEM with a reduced amount of HEPES (50 mM), 2 % FCS, penicillin (100 U/ml), and streptomycin (100 μg/ml), pH 8.2) which generates alkaline stress on the cells. Samples were incubated at 37° C without CO_2_ and the medium was changed daily. After 5 days, the cells were fixed with an ice-cold mixture of ethanol and acetone (1:1) for 15 min at room temperature. The fixative was removed and cells were washed three times with PBS and stored in PBS containing 0.1 % (w/v) bovine serum albumin (BSA) at 4 °C. To identify bradyzoite cysts, an immunostaining of the cells was performed. All incubation steps were performed at room temperature in a humidity chamber. The cells were washed with PBS once and the primary antibody added (*T. gondii* tachyzoites, polyclonal from rabbit) diluted 1:1000 in PBS with 0.01% BSA and incubated for 1 h. After washing the cells three times with PBS, an FITC-conjugated secondary antibody (polyclonal swine anti-rabbit IgG-FITC, Dako) was diluted 1:1000 together with rhodamine-conjugated *Dolichos biflorus* agglutinin (DBA-Rh, Biozol) to stain the cyst walls, diluted 1:100 in PBS with 0.01% BSA. After adding that mix to the cells, they were incubated for 1 h. Cells were washed three times with PBS and the nuclei were counterstained with DAPI (4′,6-diamidino-2-phenylindole) for 5 min. Finally, the cells were washed with PBS, covered in PBS with 0.01% BSA, and analyzed under a Leica CTR 4000 fluorescence microscope. Bradyzoite cysts in the cell layer were identified as structures with stained *T. gondii* (FITC) encased by a cyst wall (rhodamine).

### T. gondii immunization and challenge experiment

All animal experiments were carried out in accordance with the EU Directive 2010/63/EU for animal experiments and were approved by local authorities (No.: TVV 62/21). Female BALB/c mice (6 weeks old) were obtained from Charles River (Sulzfeld, Germany) and kept in a pathogen-free environment in isolated ventilated cages. The immunization and challenge dose as well as the schedule were based on a study by Hiramoto et al. (2002). Groups of 10 mice were immunized by intraperitoneal (i.p.) injection with three doses of 3 × 10^6^ tachyzoites per mouse in a bi-weekly interval. The tachyzoites (ME49 strain) were either treated with 0.25 mA or 0.1 mA LEEI or were formalin-fixed. Control mice were mock immunized with buffer solution (PBS). Blood was collected prior to the first and 1 week after each immunization. To obtain serum for analysis of *T. gondii*-specific antibodies, the samples were incubated at room temperature for 30 min and centrifuged at 8000 × g for 10 min. Two weeks after the third immunization, the mice were challenged by i.p. injection of 1 × 10^3^ tachyzoites of *T. gondii* strain RH-GFP and scored daily over 4 weeks. Clinical symptoms of disease were assessed by the following criteria: weight loss, fur condition, eye appearance, body posture, activity level, and motor function and neurological symptoms. Upon reaching the humane endpoint, mice were immediately euthanized in a CO_2_ chamber.

### Statistical analysis

Statistical analysis was conducted with GraphPad Prism 6.0.7 (GraphPad Software, Inc., La Jolla, USA). ELISA data and data for survival were analyzed by a Kruskal-Wallis test followed by a Dunn’s post hoc multiple comparison test. Data of *T. gondii*-binding antibodies were tested for normality using the Shapiro-Wilk test. Normally distributed data were consecutively analyzed using an unpaired *t*-test while not normally distributed data were analyzed using a Mann-Whitney *U* test. Level of significance is indicated with * = *p* < 0.05, ** = *p* < 0.01, *** = *p* < 0.001, and **** = *p* < 0.0001.

## Results

### Distribution of liquids in the microfluidic chip (MFC)

Uniform irradiation of samples in the MFC requires a constant flow rate and homogeneous distribution of liquid in all eight parallel flow channels. Therefore, the maximum flow velocities in the individual channels were investigated experimentally with fluorescent particles mimicking cells.

**Figure 2:**
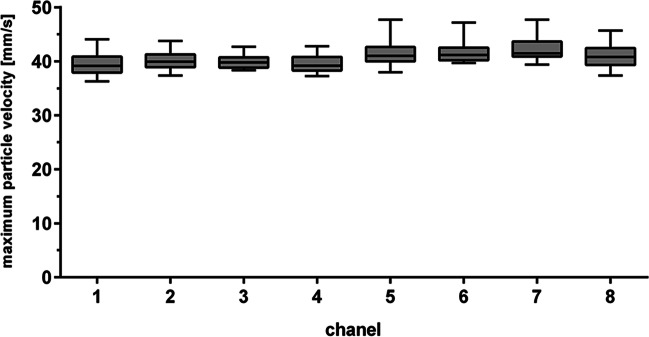
Maximum particle velocities measured in the single parallel channels of the microfluidic chip (MFC). Maximum flow velocities were measured simultaneously in the eight channels of the MFC using fluorescent tracer particles (*n* = 20)

No significant differences between the maximum particle velocities were measured in all eight parallel channels (Fig. 2).

### LEEI treatment of Cryptosporidium parvum oocysts

Different parameters of LEEI were tested on *C. parvum* oocysts in order to identify a reproducible setup for attenuation. In addition, oocysts were completely inactivated by heat and served as controls for attenuation. Excystation, intracellular replication, and antigenic conservation were studied.

**Figure 3:**
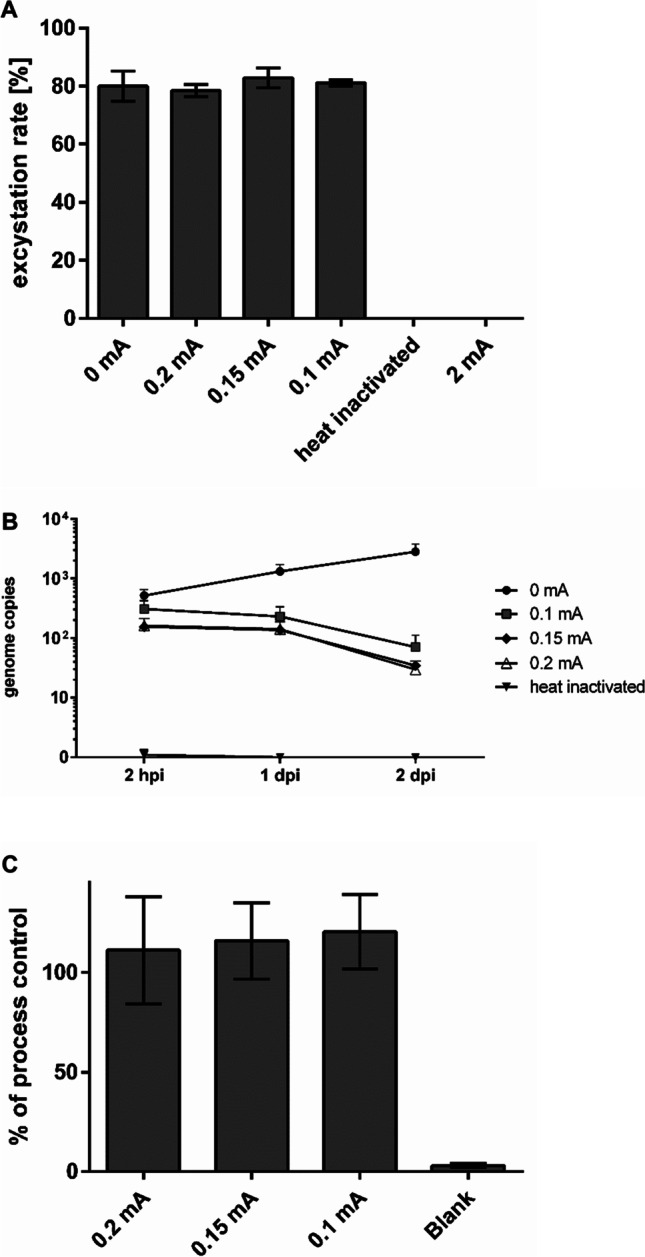
Effect of LEEI on *C. parvum* oocysts. **A** Excystation rates of oocysts after LEEI aiming for attenuation. Excystation rates in % (number of empty oocysts after release of sporozoites compared to total oocyst number per ml sample) 2 h after induction of excystation with sodium taurocholate. Data from 2 independent irradiation experiments. Samples were treated with LEEI at the indicated currents or heat-inactivated. No statistically significant differences were detected among the groups. **B** Intracellular replication of sporozoites after LEEI. Quantification of *C. parvum* DNA with real-time PCR in DNA isolated from HCT-8 cells infected with sporozoites after excystation. DNA was isolated from infected cells 2 hpi, or 1 and 2 dpi. Data from 2 independent irradiation experiments. DNA samples isolated from 2 cell culture wells per time point and each sample measured as duplicates in real-time PCR. **C** Antigenicity of oocysts after LEEI treatment. Analysis of surface proteins in an ELISA assay tested with a polyclonal antibody against *C. parvum* oocysts. Shown are mean values of samples from 4 independent irradiation experiments, each sample set analyzed in 2 independent measurements in triplicates ± standard deviations. The 0 mA control was set to 100 %. No statistically significant differences were detected among the groups

The excystation rate describes the oocysts’ ability to release infectious sporozoites and was determined after treatment with LEEI (Fig. 3A). Two hours after inducing excystation, no significant changes in excystation rates of non-irradiated oocysts and oocysts treated with 0.1, 0.15, or 0.2 mA were detected. In contrast, heat-inactivated oocysts and samples treated with LEEI at 2 mA could no longer excystate.

The radiation dose applied by LEEI is controllable by adjusting the electric current (Fertey et al. 2020). After dosimetric analysis, the amperage values of 0.1, 0.15, 0.2, and 2 mA corresponded to 1.4, 2.3, 2.9, and 25 kGy, respectively.

Intracellular replication of *C. parvum* sporozoites in HCT-8 cells was monitored via measuring intracellular genome copies by qPCR. The changes in the number of *C. parvum* genome copies in infected cells over the course of 2 dpi are shown in Fig. 3B. In the 0-mA control, the genome numbers increased steadily until 2 dpi. The initial count of genome copies at 2 hpi in LEEI-treated samples (0.1-0.2 mA) was similar to the control with only a slight decrease after 1 dpi. The numbers further declined after 2 dpi. No *C. parvum* genomes could be detected in the heat-inactivated control. In all samples, the numbers of genomes decreased after 3 dpi, including the non-irradiated control (data not shown).

The effects of LEEI on antigen structures of *C. parvum* oocysts were analyzed in an ELISA assay using a polyclonal *C. parvum* antibody. Oocysts irradiated with 0.2, 0.15, and 0.1 mA were compared to a 0-mA control (set to 100 %) (Fig. 3C). No significant differences between the applied amperages could be detected.

### LEEI treatment of Toxoplasma gondii tachyzoites

**Figure 4:**
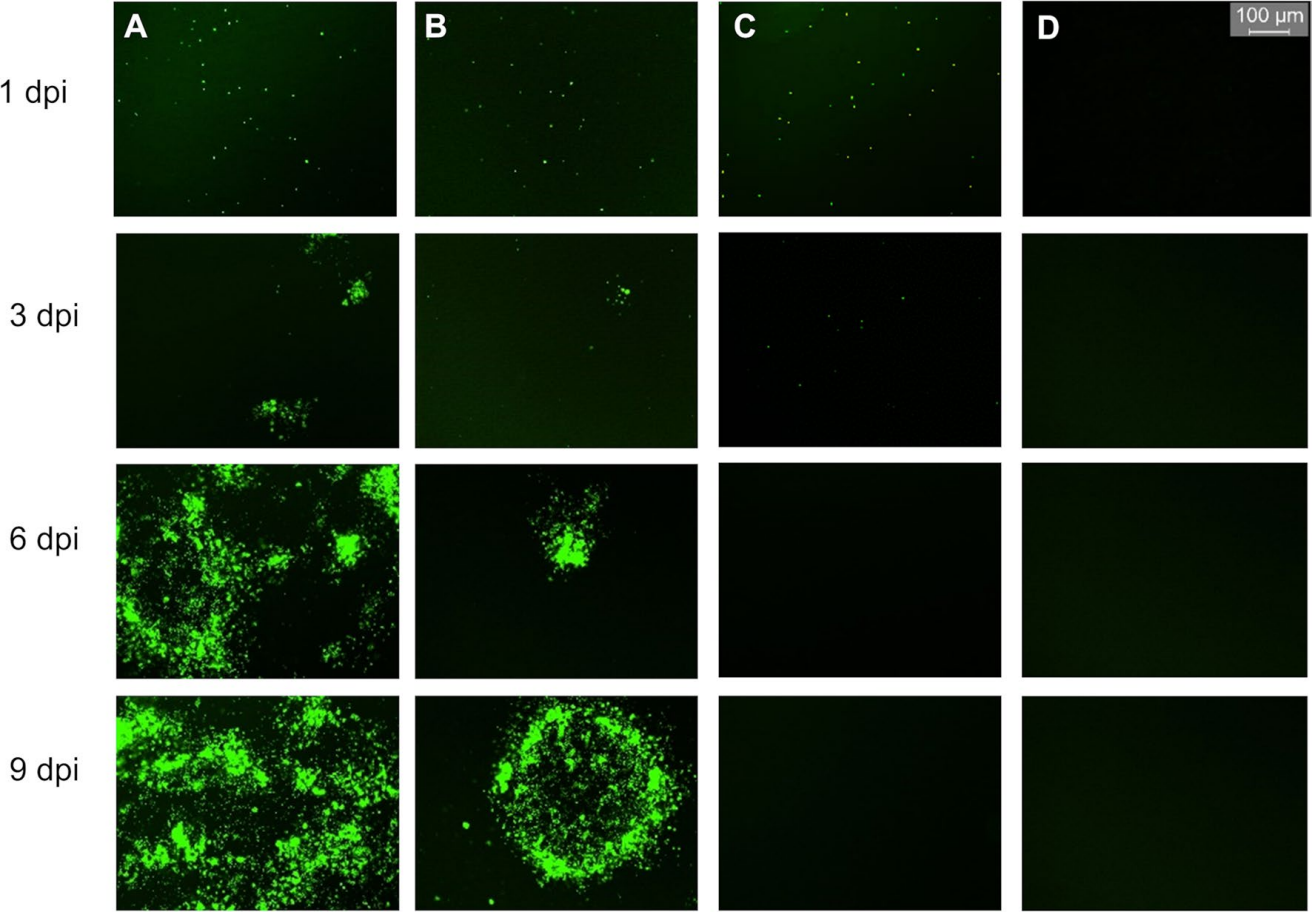
Reproductive capacity of *T. gondii* RH-GFP after LEEI treatment. Vero E6 cells infected with GFP-expressing tachyzoites after treatment with LEEI of different amperage. Cultures were observed under a fluorescence microscope for 9 days post infection (dpi). **A** Non-irradiated control. **B** Irradiated with 0.1 mA. **C** Irradiated with 0.25 mA. **D** Non-infected cells

The reproductive capacity of LEEI-treated tachyzoites of the RH and ME49 strains in vitro was analyzed with infection assays in Vero E6 cells. For a visual assessment of *T. gondii*, a GFP-marked RH strain was used. Upon LEEI treatment, cell layers were observed under a fluorescence microscope for 9 days post infection (dpi) (Fig. 4). Non-irradiated tachyzoites started replicating early after infection, and first intracellular clusters of tachyzoites were visible after 3 dpi (Fig. 4A). At 9 dpi, most of the host cells were destroyed and tachyzoites were released into the supernatant. LEEI treatment caused an amperage-dependent reduction in reproduction. All samples irradiated with 0.1 or 0.25 mA were able to infect cells, as demonstrated by singular green spots corresponding to live tachyzoites (Fig. 4B, C). Parasites irradiated with 0.1 mA were still able to reproduce in infected cells but showed a delay of 3-4 days and fewer tachyzoite clusters distributed over the cell layer compared to the non-irradiated controls. In cells infected with the 0.25 mA-treated sample, single-tachyzoite spots were visible up until 3 dpi but without further replication.

**Figure 5:**
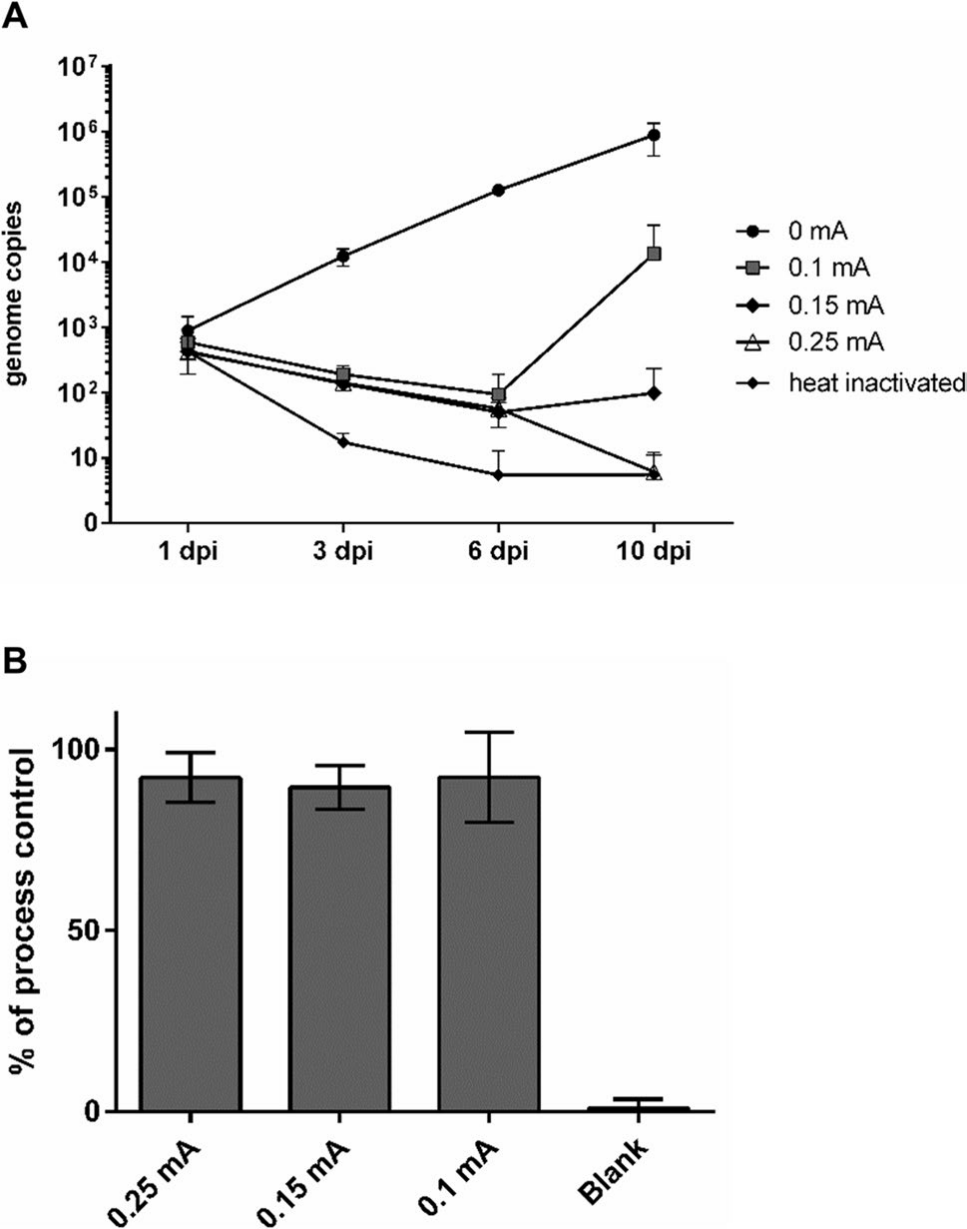
Effect of LEEI on *T. gondii* ME49 tachyzoites. **A** Intracellular replication of *T. gondii* after LEEI. Quantification of *T. gondii* DNA with real-time PCR in DNA isolated from infected Vero E6 cells. Data were obtained from 2 independent irradiation experiments. DNA samples isolated from 2 cell culture wells per time point. Each sample was measured in duplicates. **B** Antigenicity of tachyzoites after LEEI treatment. Analysis of surface proteins in an ELISA assay tested with a polyclonal antibody against tachyzoites of *T. gondii*. Shown are mean values of samples from 4 independent irradiation experiment, each sample set analyzed in 2 independent measurements in triplicates ± standard deviations *(*n= 24). The 0-mA sample (process control) was set to 100 %

We additionally analyzed intracellular replication of LEEI-treated tachyzoites of *T. gondii* strain ME49 in a qPCR assay using DNA isolated from infected Vero E6 cells. Figure 5A shows changes in the number of *T. gondii* genomes in cells over the course of 10 dpi. During that time, genome copies in cells infected with the non-irradiated process control (0 mA) steadily increased. The data of a completely untreated control were almost identical (data not shown), indicating that the microfluidic process itself (without irradiation) has no significant effect on reproduction of *T. gondii* tachyzoites. In cells infected with LEEI-treated tachyzoites (0.1-0.25 mA), the number of genomes at 1 dpi was similar to the control, but decreased in all samples until 6 dpi. At 10 dpi, the numbers of the 0.15 mA and 0.1 mA sample both increased, with a larger increase in the latter sample. After infection with the 0.25-mA sample, no increase in the genome number could be detected.

For LEEI on *T. gondii* tachyzoites, which are known to be far more sensitive to ionizing radiation than *C. parvum* oocysts, the electron flux had to be reduced mechanically (see the “Methods” section). As the dosimetric system used in the study is still under development for such lower doses, the Gy values were calculated from the higher-dose measurements applied for *C. parvum*, and are therefore approximate values: 0.1 mA, 0.15 mA, and 0.25 mA correspond to approx. 70 Gy, 115 Gy, and 185 Gy, respectively.

The effect of LEEI on antigenicity of *T. gondii* tachyzoites was analyzed in an ELISA assay. Samples irradiated with 0.25, 0.15, and 0.1 mA were compared to the 0 mA control (set to 100 %) (Fig. 5B). A polyclonal *T. gondii* antibody was used to examine the integrity of surface proteins. A minor reduction of antigenicity was detected in all samples after LEEI treatment compared to the control with no significant differences between the applied amperages (0.25 mA: 92.2 ± 6.9 %; 0.15 mA: 89.5 ± 6 %; 0.1 mA: 92.2 ± 12.4 %).

**Figure 6:**
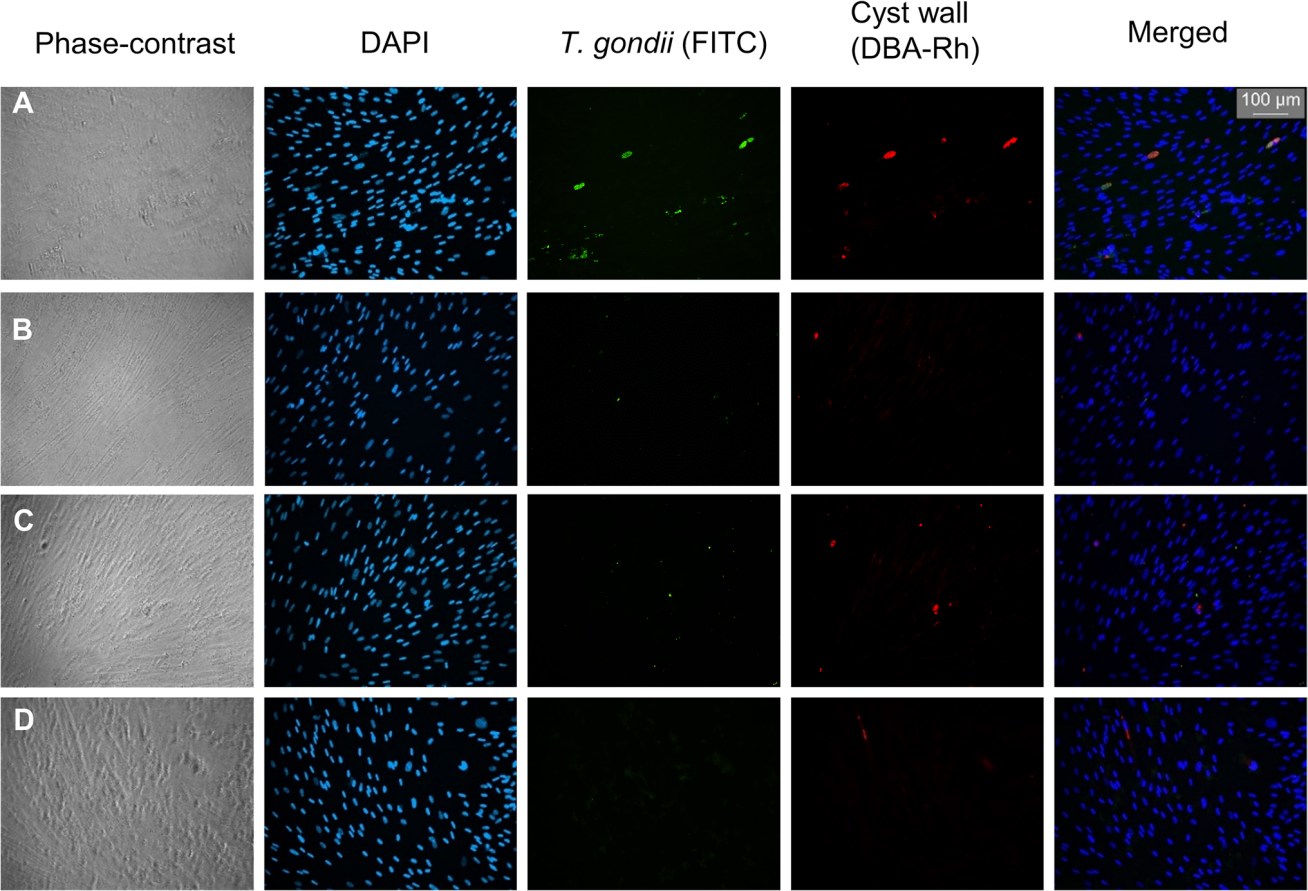
Bradyzoite cyst development of LEEI tachyzoites of *T. gondii* ME49. HFF cells infected with tachyzoites after LEEI treatment and bradyzoite differentiation induced by alkaline stress. Cells were fixed 5 days post infection (dpi). Immunostaining of *T. gondii* was performed with a polyclonal antibody and FITC-conjugated secondary antibody (green) and of bradyzoite cyst walls with rhodamine-conjugated *Dolichos biflorus* agglutinin (DBA-Rh) (red). **A** Non-irradiated control. Cysts are identified as overlapping structures in the FITC and DBA staining. **B** and **C** Irradiation with **B** 0.1 mA and **C** 0.25 mA. **D** Non-infected cells. Green structures that were not encased by a cyst wall were regarded as single tachyzoites

Bradyzoite cysts in an HFF-cell layer were analyzed by immunostaining. Figure 6 shows representative pictures of stained cells infected with *T. gondii* ME49 tachyzoites after LEEI compared to the non-irradiated control. Cysts were identified in the non-irradiated controls at 5 dpi by overlaying the staining of *T. gondii* (green) and the cyst wall (red). In 3 independent irradiation experiments, no bradyzoite cysts could be found in cells infected with the LEEI-treated samples after 5 dpi.

### Protective capacity of LEEI-treated T. gondii tachyzoites in vivo

**Figure 7:**
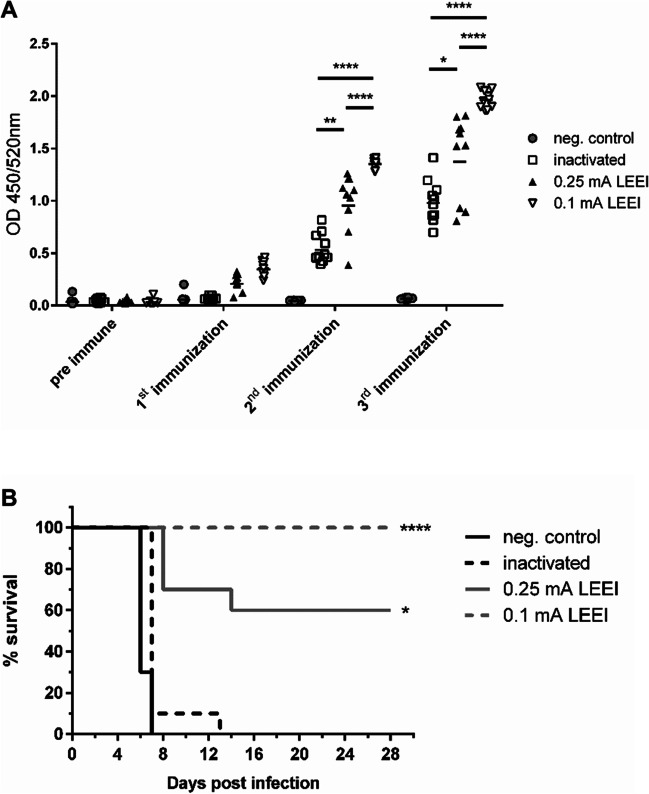
Immunization of Balb/c mice with LEEI-treated tachyzoites of *T. gondii*. **A** Antibody levels in mice (10 per group) immunized with different preparations of *T. gondii* tachyzoites (ME49 strain). Blood was sampled before the first immunization (pre-immune) and after each immunization and analyzed for IgG antibodies against *T. gondii* tachyzoites by ELISA. **B** Protection from acute infection with *T. gondii* RH-GFP. Mice, immunized with either buffer, formaldehyde-inactivated, or LEEI (0.1 or 0.25 mA) irradiated tachyzoites, were infected with tachyzoites from the RH-GFP strain. Survival is indicated by the respective lines. Statistical analysis of binding antibodies was performed by an unpaired Mann-Whitney *U* test. Survival data were analyzed using Kruskal-Wallis test and Dunn’s pairwise multiple comparison test (∗*p* < 0.05; ∗∗*p* < 0.01; ∗∗∗*p* < 0.001)

To investigate whether *T. gondii* tachyzoites attenuated by LEEI are capable of inducing protective immune responses, we analyzed them in a murine infection model. Mice were immunized three times with LEEI-treated tachyzoites from the ME49 strain (0.1 and 0.25 mA). In addition, parasites inactivated with formaldehyde were taken along as a control for complete inactivation (Krahenbuhl et al. 1972). Mice immunized with buffer only served as negative/mock controls. The application of the irradiated parasites was well tolerated by all animals, with no detectable impact on health or behavior. Antibodies (IgG) against *T. gondii* were detectable after the first immunization in the animals immunized with LEEI-treated parasites, and the titers in all groups except the negative controls increased after the second and third administration (Fig. 7A). The animals that received tachyzoites irradiated with 0.1 mA displayed the highest antibody levels, with only low variability within the group. In contrast, antibody levels in the groups immunized with parasites irradiated with 0.25 mA or treated with formaldehyde were lower and more heterogeneous.

To analyze protection, we used the RH-GFP strain to induce an acute and lethal infection (da Costa et al. 2018; Hiramoto et al. 2002), and the survival of the immunized animals was monitored. The control group and the one immunized with formaldehyde-inactivated tachyzoites showed strong symptoms starting 5 days post infection (dpi) and did not survive the first week, except one animal in the formaldehyde group that had to be euthanized 13 dpi (Fig. 7B). In the group immunized with 0.25 mA irradiated parasites, three animals had to be euthanized at 8 dpi. These were the ones showing the lowest antibody titers in the group (Fig. 7A, black triangles). One more animal in this group had to be euthanized on 14 dpi, whereas the others survived. All mice in the group immunized with 0.1 mA irradiated tachyzoites survived the infection without detectable symptoms.

## Discussion

Methods to attenuate unicellular parasites for the development of vaccines include serial in vitro or in vivo passages or the deletion of genes essential for replication (Liang et al. 2020; Minor 2015; Verma and Khanna 2013; Wang et al., 2020; Wu et al. 2022; Yang et al., 2020). As an alternative to these rather laborious and time-consuming approaches, ionizing radiation such as either gamma rays or X-rays has been used. The advantages of ionizing radiation include the short process time and the applicability to various parasite strains or isolates. Irradiation-attenuated parasites have been shown to induce protective immune responses in several animal species (Hiramoto et al. 2002; James et al. 2022; Jenkins et al. 2004; Oneko et al. 2021; Zorgi et al. 2011). On the other hand, the relatively high doses needed for irradiation of many parasites require complex irradiation facilities. Here, we demonstrate attenuation of parasites by LEEI as a method for ionizing radiation. LEEI has the advantage that it is applicable in normal laboratories or in biopharmaceutical production processes due to only minimal shielding requirements. In addition, inactivation is much faster than with current technologies, as LEEI acts within milliseconds, and the irradiation conditions are precisely controllable and reproducible (Fertey et al. 2020). Previous work demonstrated the proof-of-concept of LEEI to generate an experimental vaccine against *Eimeria tenella*; however, this was performed in a preliminary microliter scale and in a non-automated setup with no possibility for upscaling (Thabet et al. 2019). Therefore, in the present study, we use a microfluidic-based technology which represents an automated system to generate the thin liquid stream required to be fully penetrated by the low-energy electrons. It is a continuous process that can be scaled up substantially, e.g., by using more channels for the liquid and/or greater volumes of sample reservoirs. The data showed a high reproducibility of the flow velocity and a homogeneous distribution of fluids in the microfluidic channels, thus enabling an even application of exact LEEI doses during the process.

LEEI conditions for *C. parvum* and *T. gondii* were established that reproducibly caused delay or incapability to grow in vitro. In accordance to data of previous studies with LEEI-treated pathogens (Bayer et al. 2018; Fertey et al. 2020; Finkensieper et al. 2022), the antigenicity of *C. parvum* and *T. gondii* was not considerably changed during the irradiation process compared to non-irradiated controls. Our in vitro data also indicate that LEEI-treated *T. gondii* was not able to differentiate from its tachyzoite stage to bradyzoite cysts. This would minimize the risk of untreatable tissue cyst formation upon in vivo application of attenuated, but still active, parasites (Holland and Doggett 2017).

The doses delivered by low-energy electrons correlate to the electron current and can be set accordingly (Fertey et al. 2020). The oocyst stage of *C. parvum* is known to be extremely resistant to ionizing radiation, with inactivation doses between 25 and 50 kGy (Joung et al. 2011; Yoon and Yu 2011; Yu and Park 2003). In our study, oocysts treated with LEEI of up to 2.9 kGy were able to release infectious sporozoites during excystation with a rate similar to the controls, but the parasites showed no intracellular replication. This is in accordance with another study (Kato et al. 2001), where the excystation rate after 2 kGy gamma-irradiation was the same as for non-irradiated oocysts and decreased to 50% after treatment with 20 kGy. Jenkins et al. (2004) showed that immunizing cows with *C. parvum* oocysts treated with 400 Gy gamma-irradiation resulted in reduced shedding and loss of clinical symptoms upon challenge. However, the study reported a much greater impact of the irradiation on excystation rates. Different experimental setups such as incubation times and chemicals used to induce excystation might explain these discrepancies. For attenuating *T. gondii*, lower irradiation doses are needed than for *C. parvum*, and a precise dosimetric system in this dose range is still under development. The Gy values are therefore approximations. Tachyzoites treated with LEEI at a dose of approx. 185 Gy (0.25 mA) retained the ability to invade cells but failed to replicate intracellularly, which is similar to the results of previous studies using 200 or 255 Gy of gamma-irradiation (Hiramoto et al. 2002; Zorgi et al. 2011).

When injected into mice for immunization experiments, LEEI-treated tachyzoites were well tolerated, induced no clinical signs of infection, and elicited robust antibody responses against *T. gondii*. The IgG levels were higher in the animals immunized with lower-dose irradiated parasites, indicating that less attenuation led to stronger immune responses. This could be explained by a stronger stimulation of the immune system through longer residual activity of the pathogens. Similarly, in the lethal model for an acute infection, the 0.1 mA-treated tachyzoites induced complete protection, whereas survival was 60% in the group immunized with the 0.25 mA irradiated parasites. On the other hand, all mice immunized with formaldehyde-inactivated tachyzoites succumbed to the infection. In a previous study using tachyzoites that were gamma-irradiated with 200 Gy, no animals survived the challenge with the RH strain, although protection lasted longer than in the controls (Hiramoto et al. 2002). Similarly, tachyzoites irradiated with 255 Gy of gamma rays induced only partial protection against different *T. gondii* strains (Zorgi et al. 2011). The present results confirm that the irradiation dose must be selected carefully as the levels of immunogenicity and hence of protection are linked to the degree of attenuation of the parasites. In addition, safety issues of not completely inactivated parasites must be taken into account. Tachyzoites treated with LEEI of 0.1 mA did not cause detectable infection or symptoms, but induced complete protection against challenge. However, they still showed growth in cell culture, although severely delayed. To increase the safety profile of potential vaccine candidates, further fine tuning of the doses might therefore become necessary. LEEI is well suited to set precise and reproducible irradiation conditions, as the electron flux can be controlled very accurately (Fertey et al. 2020).

Current mouse models for *C. parvum* infection rely on immunocompromised animals (Dayao et al. 2022); therefore, the in vivo analysis of attenuated oocysts is challenging and still in progress.

Taken together, we present a microfluidics-based process to treat unicellular parasites with LEEI and thereby attenuate them. *Cryptosporidium parvum* and *Toxoplasma gondii*, both zoonotic pathogens with a high global impact on human and veterinary health, can be attenuated to an extent that they invade host cells but fail to proliferate. Moreover, LEEI-treated tachyzoites of *T. gondii* protect animals in a lethal infection model. This results from a solid basis for the further development of anti-parasitic vaccine candidates based on LEEI.

## Data Availability

All data generated or analyzed during this study are included in this published article.
